# 9-Isopropenyl-4-methyl-2*H*-thieno[2,3-*h*]chromen-2-one

**DOI:** 10.1107/S1600536809017930

**Published:** 2009-05-20

**Authors:** Shiqing Xu, Ying Chen, Peng Xia

**Affiliations:** a Department of Medicinal Chemistry, School of Pharmacy, Fudan University, Shanghai 200032, People’s Republic of China

## Abstract

The title compound, C_15_H_12_O_2_S, features three fused rings with a dihedral angle of 79.6 (2)° between the isopropenyl group and the thio­phene ring. In the crystal, mol­ecules are connected into a supra­molecular helical chain *via* C—H⋯O contacts.

## Related literature

The title compound was obtained unexpectedly during an attempt to prepare 4-methyl-7-(2-methyl­but-3-yn-2-ylthio)-2*H*-chromen-2-one, a key inter­mediate in our study of the synthesis of potential anti-HIV heterocyclic agents (Chen *et al.*, 2004 ▶)
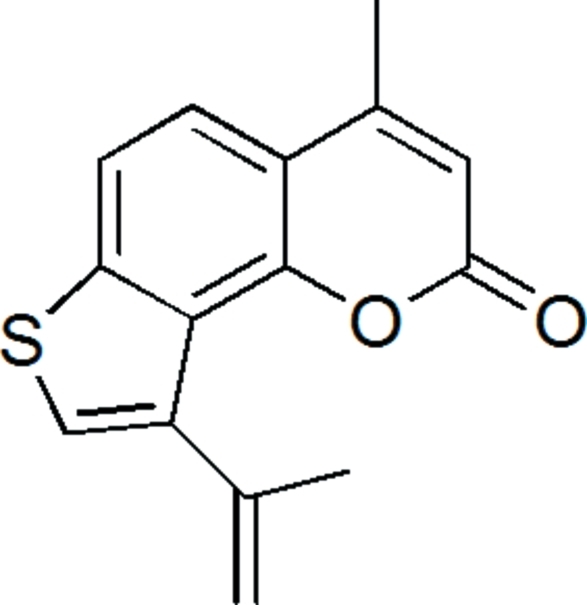


## Experimental

### 

#### Crystal data

C_15_H_12_O_2_S
*M* *_r_* = 256.31
Monoclinic, 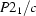

*a* = 8.547 (2) Å
*b* = 11.425 (3) Å
*c* = 13.641 (4) Å
β = 108.259 (19)°
*V* = 1265.0 (6) Å^3^
*Z* = 4
Mo *K*α radiation
μ = 0.25 mm^−1^
*T* = 293 K
0.50 × 0.15 × 0.12 mm

#### Data collection

Bruker SMART CCD area-detector diffractometer
Absorption correction: multi-scan (*SADABS*; Sheldrick, 1996 ▶) *T*_min_ = 0.887, *T*_max_ = 0.971
5816 measured reflections
2698 independent reflections
1870 reflections with *I* > 2σ(*I*)
*R*_int_ = 0.091

#### Refinement

*R*[*F* ^2^ > 2σ(*F* ^2^)] = 0.068
*wR*(*F* ^2^) = 0.177
*S* = 0.99
2698 reflections
166 parameters
H-atom parameters constrained
Δρ_max_ = 0.37 e Å^−3^
Δρ_min_ = −0.44 e Å^−3^

### 

Data collection: *SMART* (Bruker, 2000 ▶); cell refinement: *SAINT* (Bruker, 2000 ▶); data reduction: *SAINT*; program(s) used to solve structure: *SHELXS97* (Sheldrick, 2008 ▶); program(s) used to refine structure: *SHELXL97* (Sheldrick, 2008 ▶); molecular graphics: *SHELXTL* (Sheldrick, 2008 ▶); software used to prepare material for publication: *SHELXL97*.

## Supplementary Material

Crystal structure: contains datablocks I, global. DOI: 10.1107/S1600536809017930/tk2449sup1.cif

Structure factors: contains datablocks I. DOI: 10.1107/S1600536809017930/tk2449Isup2.hkl

Additional supplementary materials:  crystallographic information; 3D view; checkCIF report

## Figures and Tables

**Table 1:** Hydrogen-bond geometry (Å, °)

*D*—H⋯*A*	*D*—H	H⋯*A*	*D*⋯*A*	*D*—H⋯*A*
C11—H11⋯O2^i^	0.93	2.44	3.293 (3)	153

Symmetry code: (i) 
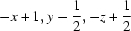
.

**Fig. 1.**
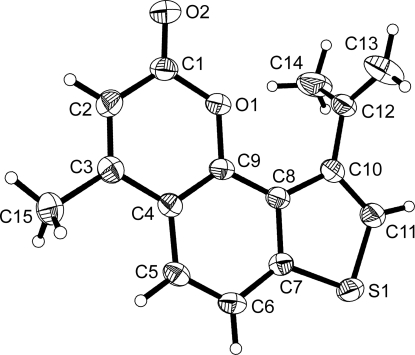
The molecular structure of (I), with atom labels and 30% probability displacement ellipsoids for non-H atoms.

## supplementary crystallographic information

### Comment

4-Methyl-7-(2-methylbut-3-yn-2-ylthio)-2*H*-chromen-2-one is a key intermediate in our study on synthesizing potential anti-HIV heterocyclic agents (Chen *et al.*, 2004). We attempted to prepare this by the reaction of 7-mercapto-4-methyl-2*H*-chromen-2-one with 2-methylbut-3-yn-2-ol in the presence of a catalytic amount of *p*-toluenesulphonate in refluxing toluene. To our surprise, an unexpected product (I) was obtained, which was structurally characterized by ^1^H NMR, MS, HRMS. The molecular structure was confirmed by X-ray diffraction. The determination of the structure of (I) is important for discovering the mechanism of this unexpected reaction. The full structural details of (I) are reported herein.

The molecular structure of (I), Fig. 1, shows three fused rings. The C10–C11 and C12-C13 bond distances of 1.351 (3) and 1.328 (4) Å, respectively indicate typical C=C double bonds. The isopropenyl group and the thiophene ring make a dihedral angle of 79.6 (2)°, due to the charge repulsion between π-electrons of isopropenyl group and the lactone-oxygen lone pair of electron lactone as well as unfavourable steric interactions. The molecular packing is stabilized by C—H···O contacts that link molecules into a supramolecular helical chain along the b-axis (Table 1).

### Experimental

A mixture of 7-mercapto-4-methyl-2*H*-chromen-2-one (961 mg, 5 mmol), 2-methylbut-3-yn-2-ol (6 ml, 60 mmol) and pyridinium *p*-toluenesulphonate (90 mg, 0.36 mmol) in dry toluene (30 ml) was refluxed for 6 h. After the toluene was evaporated in vacuo, the residue was purified by silica gel column chromatography (petroleum ether / acetone = 15 / 1) to afford 260 mg of (I); yield 21.3%. Recrystallization from isopropyl ether gave colourless crystals.

### Refinement

All H atoms were placed in the idealized positions with C—H = 0.93— 0.96 Å, and with *U*_iso_(H) = 1.2—1.5 *U*_eq_(C).

### Crystal data

**Table:**

C_15_H_12_O_2_S	*F*(000) = 536
*M**_r_* = 256.31	*D*_x_ = 1.346 Mg m^−^^3^
Monoclinic, *P*2_1_/*c*	Mo *K*α radiation, λ = 0.71073 Å
Hall symbol: -P 2ybc	Cell parameters from 812 reflections
*a* = 8.547 (2) Å	θ = 3.1–27.4°
*b* = 11.425 (3) Å	µ = 0.25 mm^−^^1^
*c* = 13.641 (4) Å	*T* = 293 K
β = 108.259 (19)°	Block, yellow
*V* = 1265.0 (6) Å^3^	0.50 × 0.15 × 0.12 mm
*Z* = 4	

### Data collection

**Table:**

Bruker SMART CCD area-detector diffractometer	2698 independent reflections
Radiation source: fine-focus sealed tube	1870 reflections with *I* > 2σ(*I*)
graphite	*R*_int_ = 0.091
φ and ω scans	θ_max_ = 27.0°, θ_min_ = 2.4°
Absorption correction: multi-scan (*SADABS*; Sheldrick, 1996)	*h* = −10→10
*T*_min_ = 0.887, *T*_max_ = 0.971	*k* = −11→14
5816 measured reflections	*l* = −14→17

### Refinement

**Table:**

Refinement on *F*^2^	Secondary atom site location: difference Fourier map
Least-squares matrix: full	Hydrogen site location: inferred from neighbouring sites
*R*[*F*^2^ > 2σ(*F*^2^)] = 0.068	H-atom parameters constrained
*wR*(*F*^2^) = 0.177	*w* = 1/[σ^2^(*F*_o_^2^) + (0.1022*P*)^2^*P*] where *P* = (*F*_o_^2^ + 2*F*_c_^2^)/3
*S* = 0.99	(Δ/σ)_max_ < 0.001
2698 reflections	Δρ_max_ = 0.37 e Å^−^^3^
166 parameters	Δρ_min_ = −0.44 e Å^−^^3^
0 restraints	Extinction correction: *SHELXL97* (Sheldrick, 2008), Fc^*^=kFc[1+0.001xFc^2^λ^3^/sin(2θ)]^-1/4^
Primary atom site location: structure-invariant direct methods	Extinction coefficient: 0.021 (5)

### Special details

**Table:**

Geometry. All e.s.d.'s (except the e.s.d. in the dihedral angle between two l.s. planes) are estimated using the full covariance matrix. The cell e.s.d.'s are taken into account individually in the estimation of e.s.d.'s in distances, angles and torsion angles; correlations between e.s.d.'s in cell parameters are only used when they are defined by crystal symmetry. An approximate (isotropic) treatment of cell e.s.d.'s is used for estimating e.s.d.'s involving l.s. planes.
Refinement. Refinement of *F*^2^ against ALL reflections. The weighted *R*-factor *wR* and goodness of fit *S* are based on *F*^2^, conventional *R*-factors *R* are based on *F*, with *F* set to zero for negative *F*^2^. The threshold expression of *F*^2^ > σ(*F*^2^) is used only for calculating *R*-factors(gt) *etc*. and is not relevant to the choice of reflections for refinement. *R*-factors based on *F*^2^ are statistically about twice as large as those based on *F*, and *R*- factors based on ALL data will be even larger.

### Fractional atomic coordinates and isotropic or equivalent isotropic displacement parameters (Å^2^)

**Table:**

	*x*	*y*	*z*	*U*_iso_*/*U*_eq_	
S1	0.97805 (8)	−0.09812 (6)	0.19803 (5)	0.0637 (3)	
O1	0.65111 (17)	0.10433 (13)	0.38658 (11)	0.0495 (4)	
O2	0.4812 (2)	0.18035 (18)	0.46125 (14)	0.0719 (6)	
C1	0.6213 (3)	0.1761 (2)	0.45948 (18)	0.0526 (6)	
C2	0.7579 (3)	0.2386 (2)	0.52571 (17)	0.0563 (6)	
H2	0.7401	0.2892	0.5745	0.068*	
C3	0.9108 (3)	0.22726 (19)	0.52044 (17)	0.0486 (6)	
C4	0.9398 (3)	0.14773 (18)	0.44540 (15)	0.0411 (5)	
C5	1.0960 (3)	0.12728 (19)	0.43491 (17)	0.0464 (5)	
H5	1.1872	0.1650	0.4793	0.056*	
C6	1.1173 (3)	0.0535 (2)	0.36125 (17)	0.0498 (6)	
H6	1.2213	0.0407	0.3552	0.060*	
C7	0.9796 (3)	−0.0016 (2)	0.29585 (16)	0.0457 (5)	
C8	0.8207 (2)	0.01396 (19)	0.30220 (15)	0.0431 (5)	
C9	0.8056 (3)	0.09080 (17)	0.37904 (16)	0.0415 (5)	
C10	0.6986 (3)	−0.0519 (2)	0.22558 (17)	0.0520 (6)	
C11	0.7694 (3)	−0.1131 (2)	0.1659 (2)	0.0627 (7)	
H11	0.7089	−0.1598	0.1114	0.075*	
C12	0.5195 (3)	−0.0597 (2)	0.21115 (19)	0.0587 (7)	
C13	0.4143 (4)	−0.0004 (4)	0.1349 (3)	0.1030 (12)	
H13A	0.3015	−0.0075	0.1233	0.124*	
H13B	0.4534	0.0484	0.0931	0.124*	
C14	0.4693 (4)	−0.1369 (3)	0.2794 (3)	0.0912 (10)	
H14A	0.3512	−0.1377	0.2605	0.137*	
H14B	0.5147	−0.1099	0.3492	0.137*	
H14C	0.5084	−0.2146	0.2738	0.137*	
C15	1.0528 (3)	0.2947 (2)	0.5901 (2)	0.0675 (8)	
H15A	1.0157	0.3400	0.6378	0.101*	
H15B	1.0967	0.3460	0.5496	0.101*	
H15C	1.1369	0.2412	0.6277	0.101*	

### Atomic displacement parameters (Å^2^)

**Table:**

	*U*^11^	*U*^22^	*U*^33^	*U*^12^	*U*^13^	*U*^23^
S1	0.0501 (5)	0.0779 (5)	0.0685 (5)	−0.0039 (3)	0.0261 (4)	−0.0209 (3)
O1	0.0338 (9)	0.0599 (10)	0.0531 (9)	0.0017 (7)	0.0113 (7)	−0.0063 (7)
O2	0.0453 (11)	0.0963 (14)	0.0778 (12)	0.0071 (9)	0.0246 (10)	−0.0110 (10)
C1	0.0417 (14)	0.0603 (14)	0.0555 (13)	0.0080 (11)	0.0149 (11)	0.0032 (11)
C2	0.0582 (16)	0.0567 (14)	0.0545 (13)	0.0085 (12)	0.0184 (12)	−0.0063 (11)
C3	0.0488 (14)	0.0448 (12)	0.0494 (12)	0.0008 (10)	0.0114 (11)	0.0021 (10)
C4	0.0392 (12)	0.0405 (10)	0.0426 (10)	−0.0018 (9)	0.0112 (9)	0.0036 (9)
C5	0.0368 (12)	0.0475 (11)	0.0524 (12)	−0.0077 (10)	0.0102 (10)	0.0029 (10)
C6	0.0350 (12)	0.0567 (13)	0.0595 (13)	−0.0025 (11)	0.0177 (10)	0.0033 (11)
C7	0.0408 (12)	0.0519 (12)	0.0465 (11)	−0.0004 (10)	0.0168 (10)	0.0007 (10)
C8	0.0358 (11)	0.0476 (12)	0.0450 (11)	0.0003 (9)	0.0112 (10)	0.0023 (9)
C9	0.0339 (12)	0.0459 (11)	0.0453 (11)	0.0026 (9)	0.0134 (10)	0.0059 (9)
C10	0.0409 (13)	0.0607 (14)	0.0498 (12)	0.0034 (11)	0.0078 (10)	−0.0060 (11)
C11	0.0472 (15)	0.0769 (17)	0.0603 (14)	0.0012 (12)	0.0114 (12)	−0.0201 (13)
C12	0.0366 (13)	0.0708 (16)	0.0617 (14)	0.0009 (12)	0.0054 (12)	−0.0187 (13)
C13	0.0510 (18)	0.118 (3)	0.121 (3)	0.0160 (19)	−0.0002 (18)	0.019 (2)
C14	0.0461 (17)	0.118 (3)	0.111 (2)	−0.0068 (18)	0.0273 (18)	0.012 (2)
C15	0.0649 (18)	0.0664 (16)	0.0681 (16)	−0.0130 (13)	0.0165 (14)	−0.0224 (13)

### Geometric parameters (Å, °)

**Table:**

S1—C11	1.706 (3)	C7—C8	1.399 (3)
S1—C7	1.728 (2)	C8—C9	1.404 (3)
O1—C9	1.365 (2)	C8—C10	1.437 (3)
O1—C1	1.373 (3)	C10—C11	1.351 (3)
O2—C1	1.205 (3)	C10—C12	1.484 (3)
C1—C2	1.424 (3)	C11—H11	0.9300
C2—C3	1.338 (3)	C12—C13	1.328 (4)
C2—H2	0.9300	C12—C14	1.442 (4)
C3—C4	1.447 (3)	C13—H13A	0.9300
C3—C15	1.499 (3)	C13—H13B	0.9300
C4—C9	1.381 (3)	C14—H14A	0.9600
C4—C5	1.406 (3)	C14—H14B	0.9600
C5—C6	1.367 (3)	C14—H14C	0.9600
C5—H5	0.9300	C15—H15A	0.9600
C6—C7	1.385 (3)	C15—H15B	0.9600
C6—H6	0.9300	C15—H15C	0.9600
			
C11—S1—C7	90.94 (11)	O1—C9—C8	116.39 (18)
C9—O1—C1	121.72 (17)	C4—C9—C8	122.13 (19)
O2—C1—O1	116.7 (2)	C11—C10—C8	110.4 (2)
O2—C1—C2	126.1 (2)	C11—C10—C12	121.8 (2)
O1—C1—C2	117.21 (19)	C8—C10—C12	127.7 (2)
C3—C2—C1	122.6 (2)	C10—C11—S1	115.0 (2)
C3—C2—H2	118.7	C10—C11—H11	122.5
C1—C2—H2	118.7	S1—C11—H11	122.5
C2—C3—C4	119.1 (2)	C13—C12—C14	123.5 (3)
C2—C3—C15	121.8 (2)	C13—C12—C10	119.4 (3)
C4—C3—C15	119.1 (2)	C14—C12—C10	117.0 (2)
C9—C4—C5	118.38 (19)	C12—C13—H13A	120.0
C9—C4—C3	117.90 (19)	C12—C13—H13B	120.0
C5—C4—C3	123.7 (2)	H13A—C13—H13B	120.0
C6—C5—C4	121.8 (2)	C12—C14—H14A	109.5
C6—C5—H5	119.1	C12—C14—H14B	109.5
C4—C5—H5	119.1	H14A—C14—H14B	109.5
C5—C6—C7	118.1 (2)	C12—C14—H14C	109.5
C5—C6—H6	120.9	H14A—C14—H14C	109.5
C7—C6—H6	120.9	H14B—C14—H14C	109.5
C6—C7—C8	123.2 (2)	C3—C15—H15A	109.5
C6—C7—S1	125.92 (17)	C3—C15—H15B	109.5
C8—C7—S1	110.87 (16)	H15A—C15—H15B	109.5
C7—C8—C9	116.35 (19)	C3—C15—H15C	109.5
C7—C8—C10	112.72 (19)	H15A—C15—H15C	109.5
C9—C8—C10	130.9 (2)	H15B—C15—H15C	109.5
O1—C9—C4	121.46 (18)		

### Hydrogen-bond geometry (Å, °)

**Table:**

*D*—H···*A*	*D*—H	H···*A*	*D*···*A*	*D*—H···*A*
C11—H11···O2^i^	0.93	2.44	3.293 (3)	153

Symmetry codes: (i) −*x*+1, *y*−1/2, −*z*+1/2.
